# Bardet-Biedl Syndrome With Renal, Cardiac, and Genitourinary Malformations: A Case Report

**DOI:** 10.7759/cureus.20577

**Published:** 2021-12-21

**Authors:** Madeeha Subhan Waleed, Ashok Abraham Varughese, Vineeth Amba, Radhika Pathalapati

**Affiliations:** 1 Internal Medicine, Capital Hospital, Islamabad, PAK; 2 Medicine, Pushpagiri Institute of Medical Sciences and Research Centre, Thiruvalla, IND; 3 Medicine, Rutgers Robert Wood Johnson Medical School, New Brunswick, USA; 4 Internal Medicine, Lower Bucks Hospital, Bristol, USA

**Keywords:** laurence-moon-bardet-biedl syndrome, bardet-biedl syndrome, renal pathology, mitral valve replacement, horse shoe kidney

## Abstract

Bardet-Biedl syndrome (BBS), also known as Laurence-Moon-Bardet-Biedl syndrome, is a unique autosomal recessive genetic disorder that involves multiple organ systems with an incidence under 1/100,000 in Europe and the USA. We present a case of a 27-year-old male with BBS and a past medical history of hypertension. He was diagnosed with BBS when he was a child. His physical examination showed polydactyly in the feet. His renal ultrasound showed the left kidney with a double collecting system and measured 1.9 × 6.1 × 3.6 cm and extended from the left upper quadrant to the left lower quadrant. His CT of the abdomen showed a horseshoe-shaped kidney with right moiety. Renal abnormalities in BBS have been identified recently. BBS is also associated with various cardiac manifestations such as patent ductus arteriosus, cardiomyopathies, and valvular diseases. BBS requires multidisciplinary management and a close follow-up with a nephrologist to decrease morbidity and mortality. Genetic and molecular mapping of this disorder will aid the understanding of congenital renal ciliopathies.

## Introduction

Bardet-Biedl syndrome (BBS), also known as Laurence-Moon-Bardet-Biedl syndrome, is a unique autosomal recessive genetic disorder that involves multiple organ systems with an incidence under 1/100,000 in Europe and the USA [1]. It is identified by neurocognitive decline, obesity, and renal and urogenital malformations. The most common renal manifestation associated with the disease is polycystic kidney disease [2]. The prevalence of renal abnormalities in BBS is approximately 53-82% [2]. We present a case of a 27-year-old Caucasian male with BBS, found to have a horseshoe-shaped kidney on renal ultrasonography. Significant cardiac abnormalities in isolated BBS were first reported in 1994, which included two cases of interventricular septum hypertrophy and two cases of dilated cardiomyopathy with no identifiable cause [2]. BBS is also associated with various cardiac manifestations such as patent ductus arteriosus, cardiomyopathies, and valvular diseases [3]. BBS requires multidisciplinary management and a close follow-up with a nephrologist to decrease morbidity and mortality. Genetic and molecular mapping of this disorder will aid the understanding of congenital renal ciliopathies.

## Case presentation

A 27-year-old male presented to the nephrology clinic with a past medical history of hypertension, aortic insufficiency, mitral valve prolapse, mitral regurgitation status post repair, cognitive delay, and hypospadias. He was diagnosed with BBS when he was a child. He was referred to a nephrologist because of proteinuria. His physical examination showed polydactyly in the feet. His basic metabolic panel is shown in Table 1 and urinalysis is shown in Table 2.

**Table 1:** Basal metabolic profile of the patient.

Tests	Reference range	Current value
Creatinine	0.97 mg/dL	0.76-1.27 mg/dL
Blood urea nitrogen (BUN)	13 mg/dL	6-20 mg/dL
Glomerular filtration rate	107 mL/min/1.73	>59 mL/min/1.73
Sodium	139 mmol/L	134-144 mmol/L
Potassium	4.6 mmol/L	3.5-5.2 mmol/L
Chloride	102 mmol/L	96-106 mmol/L
Carbon dioxide	23 mmol/L	20-29 mmol/L
Calcium	9.4 mg/dL	8.7-10.2 mg/dL
Glucose	87 mg/dL	65-99 mg/dL
BUN/creatinine ratio	13	9-20

**Table 2:** Urinalysis of the patient.

Tests	Reference range	Current value
Specific gravity	1.025	1.005-1.030
pH	7.0	5.0-7.5
Urine color	Yellow	Yellow
Protein	+2	Negative
Glucose	Negative	Negative
Ketones	Negative	Negative
Occult blood	Negative	Negative
Urobilinogen	1.0 EU/dL	0.2-1 EU/dL
Bilirubin	Negative	Negative
Nitrate	Negative	Negative
Urine protein/creatinine ratio - random urine sample	>200	<200

**Figure 1:**
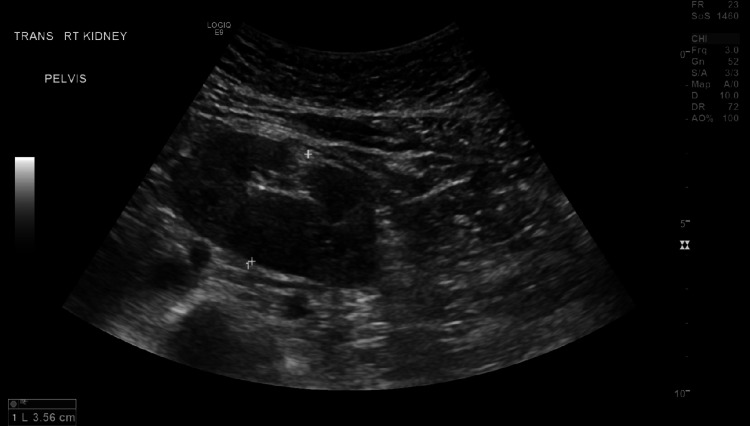
Ultrasound showing right moiety measuring 9.6 cm. There is no hydronephrosis or shadowing renal calculus. Parenchymal echogenicity is within normal limits.

**Figure 2:**
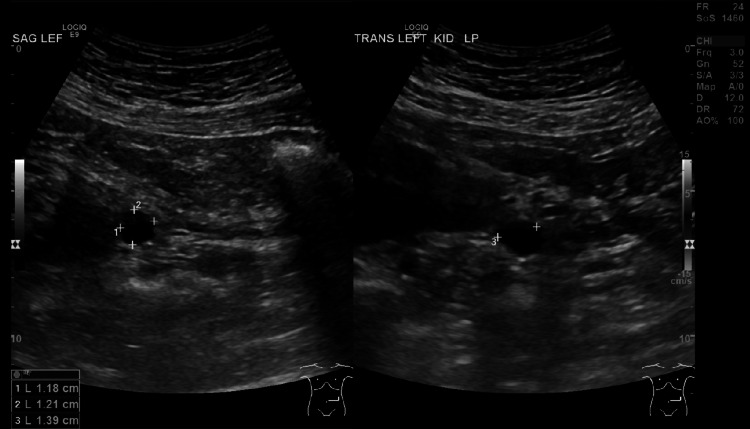
Ultrasound showing left moiety measuring 10.2 cm. There is no hydronephrosis or shadowing renal calculus. Parenchymal echogenicity is within normal limits. There is a 12 × 12 × 14 mm cyst in the lower pole of the left kidney.

His renal ultrasound revealed the left kidney with a double collecting system and measured 1.9 × 6.1 × 3.6 cm and extended from the left upper quadrant to the left lower quadrant. Left moiety measured 10.2 cm. There was no hydronephrosis or shadowing renal calculus. Parenchymal echogenicity was within normal limits. There was a 12 × 12 × 14 mm cyst in the lower pole of the left kidney. The right moiety measured 9.6 cm. There was no hydronephrosis or shadowing renal calculus. Parenchymal echogenicity was within normal limits as shown in Figures 1, 2.

His contrast tomography of the abdomen showed a horseshoe-shaped kidney with right moiety and right hepatic cyst. The rest of the presentation was unremarkable. His antinuclear antibody (ANA) titers were 1:320. The positive ANA was of unclear significance and has not changed in the past three years. He had no new positive serotypes. Proteinuria workup was unremarkable and his proteinuria resolved on the following visit six months later. The patient is currently following up with nephrology, cardiology, and geneticist. Despite the various presentations of the disease, the diagnosis can be missed because of the rarity of the condition.

## Discussion

BBS is associated with several renal malformations. Renal malformations including renal dysplasia may be present without laboratory evidence of chronic renal disease, which is likely the reason the renal manifestations of the disease go unrecognized [4,5]. Our patient also had a renal function within the age-appropriate range. In a previous cross-sectional study, the incidence of renal agenesis was found to be 1/1000 [6]. Apart from renal agenesis, other genitourinary tract malformations have been reported. Our patient did not have a right kidney. Contrarily, our patient had a horseshoe kidney abnormality. In most patients with BBS, renal insufficiency is common and subsequent renal failure is the most common cause of death [5]. Cardiovascular manifestations in BBS are fairly uncommon [7]. Our patient had a diagnosed aortic insufficiency and mitral valve prolapse, which had been surgically repaired. BBS also leads to hepatic fibrosis and biliary ductal dilation [8]. Our patient was diagnosed with a right hepatic cyst as well.

## Conclusions

BBS does not have a favorable prognosis, thus early and accurate diagnosis is required to decrease mortality and morbidity associated with the disease. Renal abnormalities are one of the leading causes of death in this syndrome. Cardiovascular risks should also be kept into consideration while treating BBS and so a multidisciplinary approach is required. Genetic testing and counseling of the family members are encouraged to facilitate the detection of genetic carriers. A better understanding of the disease pathogenesis is required to understand the correlation between the genotypic and phenotypic cardiovascular and renal manifestations of BBS.
